# SARS-CoV-2 Entry: At the Crossroads of CD147 and ACE2

**DOI:** 10.3390/cells10061434

**Published:** 2021-06-08

**Authors:** Claudio Fenizia, Silvia Galbiati, Claudia Vanetti, Riccardo Vago, Mario Clerici, Carlo Tacchetti, Tiziana Daniele

**Affiliations:** 1 Department of Pathophysiology and Transplantation, Milano University Medical School, 20122 Milano, Italy; claudio.fenizia@unimi.it (C.F.); claudia.vanetti@unimi.it (C.V.); mario.clerici@unimi.it (M.C.); 2 Department of Biomedical and Clinical Sciences “L. Sacco”, Milano University Medical School, 20157 Milano, Italy; 3 Complication of Diabetes Unit, Diabetes Research Institute, IRCCS San Raffaele Scientific Institute, 20132 Milano, Italy; galbiati.silvia@hsr.it; 4 Urological Research Institute, IRCCS San Raffaele Scientific Institute, 20132 Milano, Italy; vago.riccardo@hsr.it; 5 Faculty of Medicine and Surgery, Vita-Salute San Raffaele University, 20132 Milano, Italy; 6 IRCCS Don Carlo Gnocchi Foundation, 20162 Milano, Italy; 7 Cancer Imaging Unit, Experimental Imaging Centre, IRCCS San Raffaele Scientific Institute, 20132 Milano, Italy

**Keywords:** SARS-CoV-2, COVID-19, entry, infection, CD147, basigin, EMMPRIN, ACE2

## Abstract

In late 2019, the betacoronavirus SARS-CoV-2 was identified as the viral agent responsible for the coronavirus disease 2019 (COVID-19) pandemic. Coronaviruses Spike proteins are responsible for their ability to interact with host membrane receptors and different proteins have been identified as SARS-CoV-2 interactors, among which Angiotensin-converting enzyme 2 (ACE2), and Basigin2/EMMPRIN/CD147 (CD147). CD147 plays an important role in human immunodeficiency virus type 1, hepatitis C virus, hepatitis B virus, Kaposi’s sarcoma-associated herpesvirus, and severe acute respiratory syndrome coronavirus infections. In particular, SARS-CoV recognizes the CD147 receptor expressed on the surface of host cells by its nucleocapsid protein binding to cyclophilin A (CyPA), a ligand for CD147. However, the involvement of CD147 in SARS-CoV-2 infection is still debated. Interference with both the function (blocking antibody) and the expression (knock down) of CD147 showed that this receptor partakes in SARS-CoV-2 infection and provided additional clues on the underlying mechanism: CD147 binding to CyPA does not play a role; CD147 regulates ACE2 levels and both receptors are affected by virus infection. Altogether, these findings suggest that CD147 is involved in SARS-CoV-2 tropism and represents a possible therapeutic target to challenge COVID-19.

## 1. Introduction

In December 2019, a new member of the beta genus of coronaviruses was identified and named severe acute respiratory syndrome coronavirus 2 (SARS-CoV-2) because of its similarity to SARS-CoV [1]. Since its discovery, a lot of effort has been put in understanding how the newly identified virus enters target cells.

The spike protein has been shown to be responsible for the ability of this family of coronaviruses to interact with receptors expressed on the membrane of host cells [2]; different receptors have been identified as interactors of SARS-CoV-2 Spike protein: including Angiotensin-converting enzyme 2 (ACE2) [3], Neuropilin 1 (NRP1) [4,5], and Basigin2/EMMPRIN/CD147 (CD147) [6].

CD147, in particular, is widely expressed in human tissues and partakes in many physiological and pathological processes because of its numerous interacting partners [7]. CD147 has been reported to play an important role in human immunodeficiency virus type 1 (HIV-1), hepatitis C virus (HCV), hepatitis B virus (HBV), and Kaposi’s sarcoma-associated herpesvirus (KSHV) infections [7]. CD147 has many interactors, including secreted cyclophilin A [8], a member of the immunophilin family that also plays an essential role in promoting viral infection exerting its functions both inside and outside host cells. Thus, CyPA partakes in target cells invasion by HIV-1 and SARS-CoV [9,10], as the ability of these viruses to infect host cells depends on the interaction between CD147 and CyPA: in particular, the viral SARS-CoV nucleocapsid protein binds to CyPA, which in turn recognizes the CD147 receptor expressed on the surface of host cells [10]. At the end of 2020, Wang et al. reported for the first time the interaction between SARS-CoV-2 Spike protein and the host cell receptor CD147, and showed that modulation of the receptor levels affected the ability of the virus to infect target cells. Moreover, they showed that CD147 receptor was involved in SARS-CoV-2 infection of immune cells, which do not express ACE2, and proposed this pathway as a novel entry route [6]. Recently, the involvement of CD147 in the pathogenesis of SARS-CoV-2 infection has been questioned, as Shilts et al. reported no interaction between host cell CD147 and recombinant SARS-CoV-2 Spike protein as well as no changes in the infection ability of the virus upon knock down of CD147 in lung cells [11]. Herein we show that the CD147-CyPA complex does not play a role in SARS-CoV-2 infection, and demonstrate that silencing of CD147 reduces viral entry into pulmonary cells, either directly or indirectly via the reduction of the levels of expression of ACE2. These results indicate that the CD147 pathway of infection followed by SARS-CoV and SARS-CoV-2 is different, and suggest the potential usefulness of targeting CD147 in preventing COVID-19.

## 2. Materials and Methods

### 2.1. Antibodies and Reagents

Antibody anti-CD147 was from Santa Cruz Biotechnology (Dallas, TX, USA). Antibody anti-Spike protein antibody was from Genetex (Alton Pkwy, Irvine, CA, USA). Antibodies anti-ACE2, anti-GAPDH and anti-CyPA were from Abcam (Cambridge, UK). Blocking anti-CD147 (MEM-M6/6) antibody was from Bio-Rad Laboratories (Hercules, CA, USA). Mouse IgG were purchased from Jackson ImmunoResearch Laboratories (West Grove, PA, USA). HRP-conjugated secondary antibodies were purchased from Cell Signaling Technology (Danvers, MA, USA). Hepes, Tris, Glycine, SDS, tween 20, saponin, NH4Cl, and bovine serum albumin (BSA) were purchased from Sigma (by Merck, Kenilworth, NJ, USA). All cell culture reagents were from Thermo Scientific (Waltham, MA, USA). All chemical reagents were of analytical grade or higher, and purchased from Sigma unless otherwise specified.

### 2.2. Cell Culture, Infection, and Treatments

Vero6 (CRL-1586™, African green monkey kidney epithelial cells), A549 (CCL-185™, human epithelial cells from lung carcinoma), HepG2 (HB-8065™, human epithelial cells from liver carcinoma), CaCo2 (HTB-37™, human epithelial cells from colorectal adenocarcinoma), and CaLu3 (HTB-55™, human epithelial cells from lung adenocarcinoma) cells were purchased from American Type Culture Collection (ATCC^®^, Manassas, VA, USA). Vero E6 cells were grown in DMEM high glucose, 2 mM Glutamax, PenStrep, 10% FBS, 1 mM Hepes, and 1 mM sodium pyruvate; A549 cells were grown in DMEM high glucose, 2 mM Glutamax, PenStrep, 10% FBS, 50 uM beta-mercaptoethanol, and 1 mM sodium pyruvate; HepG2 cells were grown in DMEM high glucose, 2 mM Glutamax, PenStrep, and 10% FBS; CaCo2 cells were grown in DMEM high glucose, 4 mM Glutamax, PenStrep, 20% FBS, 1% NEAA, and 1 mM sodium pyruvate; Calu3 cells were grown in DMEM high glucose, 2 mM Glutamax, PenStrep, 10% FBS, and 1% NEAA. Cells were grown at 37 °C in 5% CO_2_ and at 98% humidity. Cells were routinely checked for mycoplasma contamination by PCR test.

CaLu3 cells were treated with 1 µg/mL anti-CD147 antibody (MEM-M6/6, Bio-rad Laboratories, Hercules, CA, USA) or IgG of mouse origin (015-000-003, Jackson ImmunoResearch Laboratories, West Grove, PA, USA) diluted in complete medium 1 h before and during infection.

In order to obtain the viral stock, SARS-CoV-2 Virus Human 2019-nCoV strain 2019-nCoV/Italy-INMI1, Rome, Italy was expanded on the human cell line CaCo2 cells and infectious viral particles concentration was assessed by TCID50 in CaLu3 cells, according to the Reed-Muench method together with the proportional distance calculation [12]. All the experiments with SARS-CoV-2 virus were performed in BSL3 facility; virus was inactivated according to institutional safety guidelines, before samples analysis outside BSL3 area.

2.5 × 10^5^ CaLu3 cells were cultured in 0.5 mL 2% FCS medium in a 24-well plate the day before. After one hour of pre-treatment with CD147-blocking Ab (or mouse IgG, as control), cells were challenged with 0.05 MOI of SARS-CoV-2. At three hpi, cells were thoroughly washed three times with pre-warmed PBS and refilled with proper growth medium (10% FCS), containing CD147-blocking Ab, mouse IgG, or plain culture medium. At 48 hpi, cells were lysed for RNA or protein extraction, whereas supernatants were harvested and appropriately stored.

### 2.3. RNA Interference

To knock down CD147 expression, specific small interfering RNA oligonucleotides (siRNA) were used [13]:

5′-GUUCUUCGUGAGUUCCUCdTdT-3′,
3′-dTdTCAAGAAGCACUCAAGGAG-5′

CaLu3 cells were transfected twice (outdistanced by 24 h) with 500 nM siRNA, either control (siRNA Negative Control Low GC Duplex #2, Thermo Scientific, Waltham, MA, USA) or against CD147, using Lipofectamine 2000 according to the manufacturer’s instructions (Thermo Scientific, Waltham, MA, USA). Transfected cells were cultured for 24 h before infection with SARS-CoV-2 and samples collected at 48 hpi.

### 2.4. RNA Extraction and Reverse Transcription

Cell supernatant was collected and Maxwell^®^ RSC Viral Total Nucleic Acid Purification Kit was used to extract RNA from 250 µL of cell culture supernatants employing the Maxwell^®^ RSC Instrument (Promega, Madison, WI, USA). The remaining supernatant was conveniently stored at −80 °C for the TCID50 assessment. Each well was then thoroughly washed three times with pre-warmed PBS. Cells were lysed and collected in 100 µL of RNAzol^®^ (TEL-TEST Inc., Friendswood, TX, USA). RNA extraction was performed employing the acid guanidium-phenol-chloroform (AGPC) extraction method, as elsewhere described [14]. One µg of total RNA was reversed transcribed in a final volume of 20 µL using the Reverse Transcription kit (Promega, Madison, WI, USA). Target cDNA was amplified by ddPCR.

### 2.5. Droplet Digital PCR (ddPCR)

The QX100™ Droplet Digital™ PCR System (Bio-Rad Laboratories, Hercules, CA, USA) instrument was used for this study. Two µL of cDNA diluted 1:10.000 (cells) or 1:100 (cell supernatants) were mixed with commercial SARS-CoV-2 (2019-nCoV) CDC qPCR Probe Assay (IDT, Coralville, IA, USA). Two µL of cDNA diluted 1:100 or 1:5 (cell extract) were mixed with commercial PrimePCR™ ddPCR™ Expression Probe Assay for CD147/BSG (Human, fluorophore FAM, dHsaCPE5045208, Bio-Rad Laboratories, Hercules, CA, USA) or ACE2 (Human, fluorophore HEX, dHsaCPE5036253, Bio-Rad Laboratories, Hercules, CA, USA), respectively. The volume of the final PCR mix was 20 µL including 10 µL of ddPCR™ Supermix for Probes (No dUTP) and 1 µL of the primers/fluorophore probe. ddPCR amplification reagents were purchased from Bio-Rad Laboratories (Hercules, CA, USA). The droplet emulsion was thermally cycled on C1000 Touch Thermal Cycler (Bio-Rad Laboratories, Hercules, CA, USA) instrument. Cycling conditions were 95 °C for 5 min, followed by 40 cycles of amplification (94 °C for 30 s and 55 °C for 1 min), ending with 98 °C for 10 min. The concentration of the target was calculated automatically by the QuantaSoft™ software version 1.7.4 (Bio-Rad Laboratories, Hercules, CA, USA). N1, ACE2, and CD147 RNA levels were normalized to total RNA. The levels of CD147-blocking antibody treated samples were normalized to those of the corresponding IgG isotype-treated control. The levels of the CD147-knocked down samples were normalized to those of control NT siRNA.

### 2.6. Western Blotting

For WB analysis cells were lysed directly in 2× Laemmli buffer in order to inactivate the virus and to be able to process them outside BSL3 area. Samples were boiled for 5 min at 95 °C before loading onto the gel. Proteins were separated by SDS–PAGE and transferred onto nitrocellulose membranes (Hybond, GE Healthcare, Chalfont St. Giles, Buckinghamshire, UK). Strips containing the proteins of interest were incubated in 5% (*w*/*v*) BSA in TBS containing 0.1% (*v*/*v*) Tween-20, pH 7.4 (T-TBS), for 1 h at room temperature and then with fresh blocking buffer containing the primary antibody at its working concentration (Table 1). After overnight incubation at 4 °C, the antibodies were removed and the strips washed with T-TBS for 3 × 10 min. Strips were incubated for 1 h with the appropriate horse radish peroxidase (HRP)-conjugated secondary antibody and washed 3 × 10 min with T-TBS. Western blots were developed using the chemiluminescent method (ECL, GE Healthcare, Chalfont St. Giles, Buckinghamshire, UK) and signals acquired by ChemiDoc MP Imaging System (Bio-rad Laboratories, Hercules, CA, USA). Bands were quantified by densitometric analysis using the National Institutes of Health (NIH) ImageJ program. The quantification of each band was normalized using the signal of GAPDH as the loading control. The signal of CD147-blocking antibody treated samples was normalized to the value of the corresponding IgG isotype-treated control. The signal of the CD147-knocked down samples was normalized to the value of control NT siRNA.

## 3. Results

Since SARS-CoV-2 is able to infect different organs, we tested four different human cell lines (A549 and CaLu3 from lungs, HepG2 from liver, and CaCo2 from intestine) as model systems to study viral infection and used Vero E6 cells from African green monkey kidney (standard system for laboratory propagation of viruses) to initially expand SARS-CoV-2. CaLu3 pulmonary cells were the most efficiently infected (in agreement with previous findings [15]), and, therefore, these cells were used as the model system for all the experiments reported here.

### 3.1. CD147 Plays a Different Role in SARS-CoV and SARS-CoV-2 Entry

SARS-CoV and SARS-CoV-2 display a high level of protein sequence homology and exploit the same protein (Spike) to enter host cells [16]. For SARS-CoV entry it has been reported that virus-associated CyPA interacts with CD147, allowing the internalization of the virus [10]. To test whether SARS-CoV-2 infection depends on the same mechanism, we treated CaLu3 cells with an anti-CD147 antibody able to block the immunophilin binding to the receptor [17] before cell incubation with the virus, and evaluated infection efficiency.

**Figure 1:**
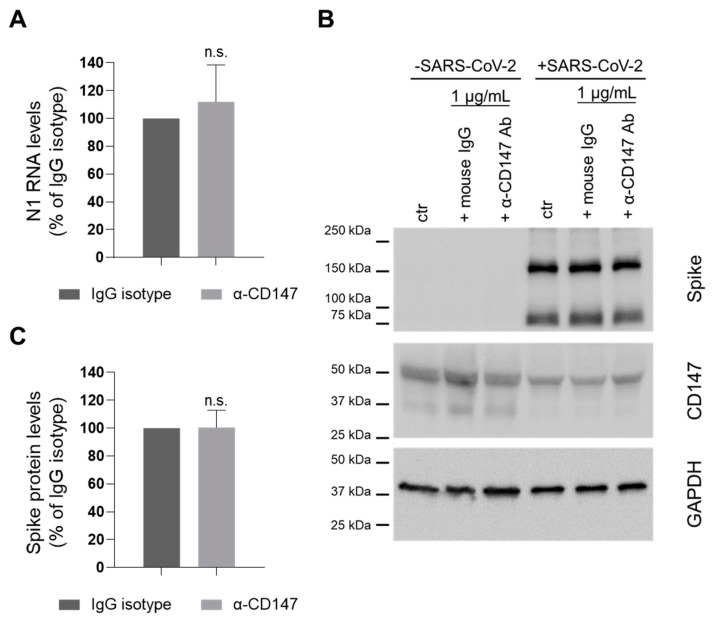
Inhibition of CyPA binding to CD147 does not play a role in SARS-CoV-2 entry. CaLu3 cells were treated with 1 µg/mL MEM-M6/6 CD147 blocking Ab (α-CD147 Ab) or equivalent mouse IgG (as isotype control) or plain medium (as control, ctr) for one hour before, and during SARS-CoV-2 infection. Samples were collected 48 hpi. (**A**) N1 RNA levels were analyzed by ddPCR and their relative abundance in cells is shown. N1 RNA levels were normalized to total RNA. N1 levels in CD147-blocking antibody treated samples were normalized to the levels in the corresponding IgG isotype-treated control. Mean ± s.e.m out of three biological replicates is shown. n.s. not significative, Student’s t test. (**B**,**C**) Lysates were analyzed by WB. (**B**) One experiment is shown as representative of three. Immunolabelling for CD147 showed that Ab treatment did not affect the levels of expression of the receptor either in the absence or in the presence of SARS-CoV-2. (**C**) Spike protein levels were normalized to GAPDH (loading control). Spike levels in CD147-blocking antibody treated samples were normalized to the levels in the corresponding IgG isotype-treated control. Mean ± s.e.m. out of three biological replicates is shown. n.s. not significant, Student’s *t*-test.

To this purpose, we performed ddPCR assay to measure nucleocapsid (N1) RNA levels (112.0 ± 26.6 percent of isotype control, Figure 1A), and WB analysis to evaluate the expression of Spike protein (96.6 ± 7.1 percent of isotype control, Figure 1B,C). Altogether, our results show that the blocking Ab did not impair SARS-CoV-2 infection ability.

These results suggest that CyPA binding to CD147 does not play a role in virus entry and that CD147 role in SARS-CoV-2 is likely different from the one in SARS-CoV infection.

### 3.2. CD147 Silencing Reduces ACE2 Levels and SARS-CoV-2 Infection

**Figure 2:**
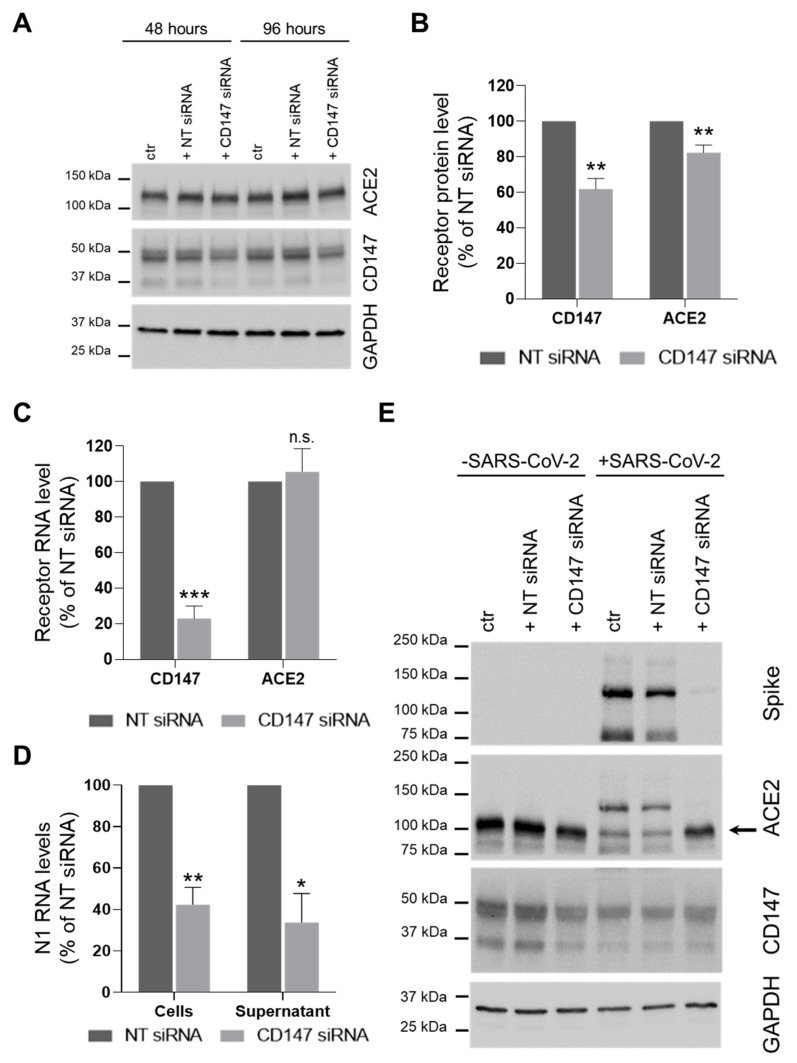
CD147 silencing reduces ACE2 protein levels and impairs SARS-CoV-2 infection. (**A**–**C**) CaLu3 cells were transfected with a non-targeting (NT) or a CD147-specific siRNA. Samples were collected 48 h and 96 h after transfection. (**A**) One experiment is shown as representative of three. (**B**,**C**) Receptor levels were analysed by WB (protein, (**B**)) and by ddPCR (RNA, (**C**)) analysis. (**B**) Receptors’ protein levels were normalized to the loading control (GAPDH). Receptors levels in CD147 siRNA-treated samples were normalized to the levels in the corresponding NT siRNA-treated controls. Mean ± s.e.m. out of three biological replicates is shown. **, *p* = 0.005 for CD147 and *p* = 0.01 for ACE2, Student’s *t*-test. (**C**) Receptors RNA levels were normalized to total RNA. Receptors levels in CD147 siRNA-treated samples were normalized to the levels in the corresponding NT siRNA-treated controls. Mean ± s.e.m. out of three biological replicates is shown. ***, *p* = 0.001, n.s. not significative, Student’s *t*-test. (**D**,**E**) CaLu3 cells were transfected with a non-targeting (NT) or a CD147-specific siRNA before SARS-CoV-2 infection. Samples were collected 48 hpi. (**D**) N1 RNA levels were analyzed by ddPCR, and their relative abundance in both cells and supernatants is shown. N1 RNA levels were normalized to total RNA. N1 levels in CD147 siRNA-treated samples were normalized to the levels in the corresponding NT siRNA-treated controls. Mean ± s.e.m. out of three biological replicates is shown. **, *p* < 0.01; *, *p* < 0.05, Student’s *t*-test. (**E**) Lysates were analyzed by WB. One experiment is shown as representative of three. ACE2 labeling was detected after Spike protein immunostaining on the same membrane. Arrow indicates ACE2 specific band. GAPDH was used as the loading control.

Since there are contradictory evidences regarding the role of CD147 in SARS-CoV-2 infection [6,11], we first decided to knock down the expression of the receptor in the physiological context of lung CaLu3 cells by siRNA transfection before infection with SARS-CoV-2 (Figure 2A–C). CD147 levels were 61.9 ± 6.0 and 32.9 ± 7.0 percent of NT siRNA-treated cells (protein and RNA, respectively). Results showed that knockdown of CD147 also reduced the abundance of ACE2 protein (82.3 ± 4.3 percent of control NT siRNA-transfected cells, Figure 2B), but not of its RNA levels (Figure 2C).

We then evaluated whether CD147 silencing affected SARS-CoV-2 infection. Results showed that viral N1 RNA levels in both cells and supernatants were reduced: 42.3 ± 8.4 and 33.7 ± 14.0 percent of control NT siRNA treated samples, respectively (Figure 2D). Furthermore, silencing of CD147 also reduced Spike protein abundance (5.0 ± 1.5 percent of control NT siRNA transfected cells, *p* < 0.01 Student’s *t*-test, Figure 2E).

Altogether, these results support previous findings reporting a role for CD147 in SARS-CoV-2 infection [6] and suggest that CD147 plays an important role in SARS-CoV-2 infection either directly or indirectly, by means of its ability to regulate ACE2 abundance at the post-translational level.

**Figure 3:**
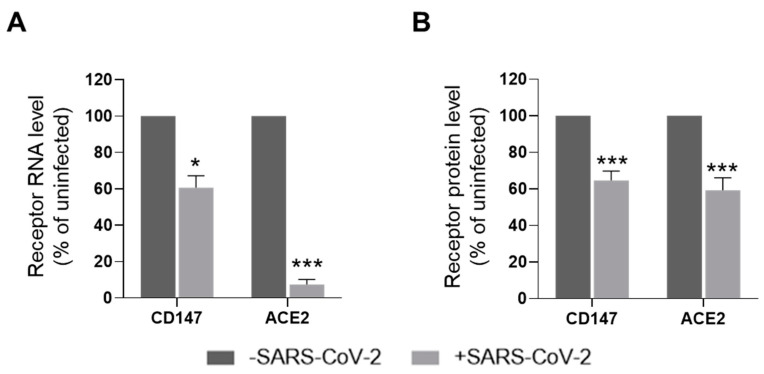
CD147 and ACE2 expression is affected by SARS-CoV-2 infection. (**A**,**B**) CaLu3 cells were infected with SARS-CoV-2 at 0.05 MOI and samples collected 48 hpi. Receptors levels were analyzed by ddPCR (RNA, (**A**)) and WB (protein, (**B**)). (**A**) Receptors RNA levels were normalized to total RNA. Receptors levels in infected samples (+SARS-CoV-2) were normalized to the levels in the corresponding uninfected controls (-SARS-CoV-2). Mean ± s.e.m. out of three biological replicates is shown. *, *p* = 0.02; ***, *p* = 0.001, Student’s *t*-test. (**B**) Receptor protein levels were normalized to the loading control. Receptor levels in infected samples (+SARS-CoV-2) were normalized to the levels in the corresponding uninfected controls (-SARS-CoV-2). Mean ± s.e.m. out of 10 biological replicates is shown. ***, *p* < 0.0001, Student’s *t*-test.

SARS-CoV entry lowers the levels of expression of ACE2 in CaLu3 cells [18,19]; thus, we investigated the effects of SARS-CoV-2 infection on receptors levels. To this purpose, we compared the levels of expression of both ACE2 and CD147 in CaLu3 cells infected with SARS-CoV-2 at 0.05 MOI and uninfected cells. At the RNA level, we found that viral infection lowers both CD147 and ACE2 (60.6 ± 6.7 and 7.3 ± 2.9 percent of uninfected cells, respectively, Figure 3A). At the protein level, we observed that SARS-CoV-2 entry decreases both CD147 and ACE2 expression (64.6 ± 5.2 and 59.3 ± 6.7 percent of uninfected cells, respectively, Figure 3B).

We finally observed that the reduction in receptor abundance induced by viral infection did not cumulate to that caused by CD147 knock down, since silenced cells were less infected by SARS-CoV-2. Thus, CD147 levels in infected ctr and NT siRNA were similar to its levels in uninfected silenced cells, and ACE2 levels in CD147-silenced cells were higher than in the infected ctr and NT siRNA-treated cells (as ACE2 reduction was higher upon viral infection than upon CD147 silencing).

Altogether, these data suggest that CD147 and ACE2 activities in SARS-CoV-2 entry are co-regulated as the expression of both receptors is downregulated upon virus exposure, indicating that viral infection acts at the transcriptional level.

## 4. Discussion

In late 2019, a new betacoronavirus was identified as the viral agent responsible for the coronavirus disease 2019 (COVID-19) pandemic. During 2020, it has become clear that this pathology affects different organs, and might induce long-lasting/persisting symptoms. Despite a huge effort and a wide number of approaches tested [20], until now no effective drugs that can inhibit SARS-CoV-2 infection have been approved. A better understanding of the molecular machinery involved in viral entry into host cells is thus of pivotal importance to explain and possibly counteract the pathological features of COVID-19 and to identify a putative target and ultimately develop effective drugs. In particular, the identification and targeting of the host factors involved in viral entry would help facing the ability of viruses to mutate to escape immune defenses and drug selective pressure.

Coronaviruses Spike protein has been shown to be responsible for their ability to interact with host membrane receptors [2] and different proteins have been identified as SARS-CoV-2 Spike protein interactors, including ACE2 [3], NRP1 [4,5], and CD147 [6].

The viremia is generally undetectable and therefore the ability of SARS-CoV-2 to invade differentially target tissues and organs might specifically depend on the relative abundance and/or balance of host receptors. CD147 is widely expressed by different cell populations and its levels of expression can be upregulated in pathological conditions. Moreover, the peculiar expression of CD147 receptor on immune cells [21] and its well-established role in inflammation (as the target receptor for secreted cyclophilins CyPA and CyPB [22]) make this receptor an interesting candidate to be investigated in the context of SARS-CoV-2 tropism and pathogenic activities.

To test its role, we knocked down the expression of the receptor by transient siRNA transfection, and found that the viral load in pulmonary cells was significantly reduced both at the RNA and protein level, while the role of CD147 in SARS-CoV-2 invasion of host cells had been excluded by Shilts et al. [11] based on experiments performed on CRISPR/Cas9 edited cells.

Because of the high sequence homology between SARS-CoV-2 and SARS-CoV, we tested the hypothesis that CD147 might be involved in SARS-CoV-2 entry by interacting with virus-associated CyPA (as already reported for SARS-CoV [10]). Pre-treatment with an antibody able to specifically block one of the several functions of CD147 (CyPA binding [17,23]), different from Meplazumab (used by Wang et al. [6]), did not affect SARS-CoV-2 entry, thus suggesting that the molecular mechanism underlying CD147 role might be different from the one reported for SARS-CoV infection.

In this context, the novel observations that SARS-CoV-2 infection reduces the expression of both ACE2 and CD147 and that silencing of CD147 decreases ACE2 expression suggest that CD147 affects virus entry into host cells either directly or indirectly via its ability to modulate ACE2 abundance.

The observation that virus entry lowers both ACE2 and CD147 RNA levels in CaLu3 cells would suggest that the two genes might be regulated in a similar manner at the transcriptional level upon infection. ACE2 has been proposed to be an interferon-stimulated gene in epithelial cells based on the analysis of primary cells treated with interferon and of surgical explant samples from healthy donors and patients with influenza A or B infections [24]. Hence, either interferon treatment by itself does not completely recapitulate the host reactions to SARS-CoV-2 infection, or SARS-CoV-2 behaves differently from influenza A and B viruses in terms of interferon host responses. Therefore, the dissection of the role of the interferon host response in SARS-CoV-2-triggered reduction of ACE2 and CD147 receptor expression remains a challenge for future studies.

CD147 has been reported to function as a chaperone for the correct trafficking of monocarboxylate transporters (MCTs) to the plasma membrane, as either its knockdown [25] or knockout [26] impairs both MCTs protein expression and their proper localization. For instance, in the absence of CD147 MCT3 redistributes from the basolateral to the entire plasma membrane of the retinal pigmented epithelium [26]. Thus, our results cannot exclude the possibility that CD147 might participate in ACE2 transport to the plasma membrane. Furthermore, CD147 has been reported to relocalize from the basolateral domain to the entire membrane of SARS-CoV-2 infected renal tubular epithelial cells [27] and suggested to contribute to virus entry from their apical membrane. Thus, we cannot exclude the possibility that ACE2 and CD147, which usually reside in different membrane domains (apical [28] and basolateral [29], respectively), localize to the same compartment upon viral invasion.

Altogether, our results support the previous finding that CD147 partakes in SARS-CoV-2 infection [6] at odds with Shilts and colleagues [11] and provide some additional clues on the underlying mechanism: (1) CD147 binding to CyPA does not play a role; (2) CD147 is involved in virus entry; (3) the two receptors might co-operate as they are co-regulated by viral infection and ACE2 levels are modulated by CD147 abundance.

CD147 belongs to the Ig superfamily, and it is expressed in several tissues, including brain, heart, liver, kidney, and immune and blood cells [30]; thus, it might play a multifaceted role in COVID-19 and possibly contribute to the worse prognosis of patients affected by comorbidities. CD147 levels are high in different organs and are upregulated in pathological conditions, including several disorders of the central nervous system [31]. Notably, CD147 has also been shown to partake in cardiovascular diseases, namely atherosclerosis and myocardial infarction [32]. CD147 is also involved in kidney diseases during both acute ischemic and chronic fibrotic injuries [33,34]. Finally a role for this receptor has been shown in pulmonary hypertension [35]. Moreover, CD147 acts both regulating target tissue homeostasis, by means of its binding to many different interactors, and modulating inflammatory processes. The wide variety of cell types expressing CD147 could explain the promising preliminary results obtained by testing the CD147-targeted antibody Meplazumab in COVID-19 patients (NCT04275245 [36]). Indeed, the possible clinical benefit observed in Meplazumab-treated patients could be explained by its direct role on SARS-CoV-2 infection as well as by its activities in patient organs where CD147 modulates tissue: (1) metabolism (binding MCTs and the amino acid transporter CD98); (2) permeability (controlling the levels and the activity of matrix metalloproteases); (3) vascularization (inducing the synthesis and the release of vascular endothelial growth factor, VEGF, and the expression of the VEGF receptor); (4) inflammation (mediating both the recruitment and the infiltration of leukocytes through its interaction with both chemokines and adhesion molecules, including integrins, selectins, CD44) [7,37].

Identifying additional cell-entry mechanisms might help the design of novel pharmacological approaches that could offer clear therapeutic advantages in the prevention of SARS-CoV-2 infection. Indeed, SARS-CoV-2 vaccines efficacy could be reduced by certain mutations and novel alternative therapeutic approaches might be essential in the clinical management of COVID-19 patients [38,39,40,41,42].

## Figures and Tables

**Table 1:** Ordering information and working conditions of used antibodies.

Antibody	Company	Catalog	Dilution	Conditions
Spike	Genetex	632,604	1:1000	Overnight, 4 °C
ACE2	Abcam	15,348	1:1000	Overnight, 4 °C
CD147	Santa Cruz	53,693	1:500	Overnight, 4 °C
CyPA	Abcam	58,144	1:500	Overnight, 4 °C
GAPDH	Abcam	128,915	1:40,000	Overnight, 4 °C
Anti-mouse HRP	Cell Signaling Technologies	7076	1:5000	1 h, r.t.
Anti-rabbit HRP	Cell Signaling Technologies	7074	1:5000	1 h, r.t.

## Data Availability

Not applicable.
